# Trends in solids/liquids poisoning suicide rates in Taiwan: a test of the substitution hypothesis

**DOI:** 10.1186/1471-2458-11-712

**Published:** 2011-09-20

**Authors:** Jin-Jia Lin, Tsung-Hsueh Lu

**Affiliations:** 1 Department of Psychiatry, Chi-Mei Medical Center, Tainan, Taiwan; 2 Department of Psychiatry, Chi-Mei Hospital, Liuying Campus, Tainan, Taiwan; 3 Department of psychiatry, School of Medicine, College of Medicine, Taipei Medical University, Taipei, Taiwan; 4 Institute of Public Health, College of Medicine, National Cheng Kung University, Tainan, Taiwan

## Abstract

**Background:**

Several previous studies have illustrated that restricting access to lethal methods can reduce suicide rates. The most often cited example was Kreitman's study, showing a reduction not only in gas-specific suicide rates, but also in the overall suicide rates because of the lack of increase of other methods. However, method substitution is still a major concern in the application of the means restriction strategy to prevent suicide. The aim of the study was to investigate whether the reduction in the solids/liquids poisoning suicide rate in 1983-1993 after the launching of pesticide restriction interventions in Taiwan was accompanied with an increase in the suicide rate using other methods (method substitution).

**Methods:**

Data on age-, sex- and method-specific suicide rates for 1971-1993 in Taiwan were obtained. Changes in solids/liquids poisoning suicide rates were compared with suicide rates by hanging and other methods between 1983 and 1993.

**Results:**

No concomitant increase in suicide rates by hanging or other methods was noted from 1983 to 1993, during which the suicide rates by poisoning with solids/liquids (mainly pesticides) decreased markedly and steadily. The phenomenon of method substitution was also not found by sex and age groups.

**Conclusion:**

In general, no method substitution was found along with the reduction in solids/liquids suicide rates in Taiwan. Our study results have also added the evidence that restricting access to methods maybe a promising strategy in preventing suicide, particularly in those countries where the "target method" has been found to contribute greatly to the suicide rates.

## Background

A reduction in suicide rates after the launching of access restriction interventions was noted in several studies of suicide methods, involving domestic gases [1-3], vehicle emissions [4], prescriptions [5,6], pesticides [7] and guns [8-11]. However, a method substitution hypothesis has been proposed, in that a compensatory increase in suicide rates by other methods might be observed. Studies revealed that domestic gas might be substituted by car exhaust [3,12], gassing might be substituted by drug overdose [13], firearms might be substituted by jumping [8] or hanging [14], and car exhaust might be substituted by hanging [15] (see Table 1). However, all previous studies were done in Western countries and pesticides were not discussed in these studies.

**Table 1:** Summary of method substitution in ecological studies

Authors	Years	Location	Target method	Substituted method	Subpopulation
Burvill et al [12]	1910-1987	Australia	Domestic gas	Car exhaust	Males
Lester [3]	1950-1970	United States	Domestic gas	Car exhaust	Males
Gunnel et al [13]	1950-1975	England	Gassing	Overdose	Women or young men
Rich et al [8]	1973-1983	Canada	Gun	Jumping	Males
Caron [14]	1986-1996	Quebec*	Gun	Hanging	The youths
Amos et al [15]	1987-1998	England	Car exhaust	Hanging	The young

* Northern Quebec (Abitibi-Temiscamingue)

It is noteworthy that dominant suicide methods differ greatly across countries, particularly between Asian and Western countries. Poisoning with pesticides was the most common suicide method used by Asian people living in rural areas (notably China, Sri Lanka, Taiwan and India) [16]. Restricting access to pesticides was strongly suggested by the World Health Organization to be a major suicide prevention strategy for Asian countries [17]. However, few studies from Asian countries have investigated whether there was method substitution once access to pesticides was restricted.

In Taiwan, poisoning with solid/liquid substances (mainly pesticides) and hanging were the two most common suicide methods, and were responsible for about 90% of all suicide deaths before 1990 [18]. The solids/liquids suicide rate dramatically decreased during the 1980s, due to the decline in the agricultural population and a series of measures launched by the government to restrict the availability of pesticides in the general population [19]. However, little is known about whether method substitution occurred in Taiwan after restricting access to pesticides.

The aim of the study was to investigate whether the reduction in the solids/liquids poisoning suicide rate in 1983-1993 after the launching of pesticide restriction interventions in Taiwan was accompanied with an increase in the suicide rate using other methods (method substitution).

## Methods

### Data sources

Using national mortality data files from the Department of Health of the Executive Yuan of Taiwan, we obtained information on age- and sex-specific annual suicide deaths from 1971 to 1993. Causes of death were categorized based on the International Classification of Diseases 8^th ^revision (ICD-8) for the years 1971-1980, and 9^th ^revision (ICD-9) for the years 1981-1993. Since suicide mortality statistics were usually underestimated, and the most common category being misclassified was that of undetermined death [20], we defined suicides as those deaths coded E950-E959, and undetermined deaths as those coded E980-E989. The ICD codes for suicide and undetermined deaths did not change from the Eighth Revision to the Ninth Revision.

### Analysis

Data were analyzed by using SPSS for Windows, Version 12.0 (SPSS Inc., Chicago, Illinois, U.S.A.). Age-, sex- and method-specific suicide rates were calculated to examine the suicide trends. Age-adjusted suicide rates were calculated using the world population as a standard. The suicide methods were grouped into three categories: poisoning with solids/liquids (E950 and E980), hanging (E953 and E983), and other methods (E951 plus E981, E952 plus E982, E954-E959 plus E984-E989). The linear regression slopes (including 95% confidence interval) for suicide rates by solid/liquids, hanging, and other methods were calculated to examine the trends of method-specific suicide rates during the period 1983-1993. Changes in method-specific suicide rates between 1983 and 1993 were also compared by sex among four age groups: 15-24, 25-44, 45-64, and 65 years and older.

Only 3-digit ICD codes were used for underlying cause of death coding in Taiwan, and some detailed information was not available. For example, the 3-digit ICD codes E950 and E980 included pesticides (E950.6 and E980.6), analgesics (E950.0 and E980.0), barbiturates (E950.1 and E980.1), other sedatives/hypnotics (E950.2 and E980.2), and other solids/liquids. Fortunately, the Department of Health of Taiwan conducted a project to retrospectively recode the underlying cause of death according to the ICD-10 for selected years. We obtained the recoded mortality data for the years 1987 and 1992, from which the solid and liquid poisoning deaths could be further classified as pesticides (X68 and Y18), medications (X60-X64 and Y10-Y14), and others (X65, X66, X69 and Y15, Y16, Y19). We compared the rate differences between 1987 and 1992 by sex and age groups.

## Results

**Figure 1:**
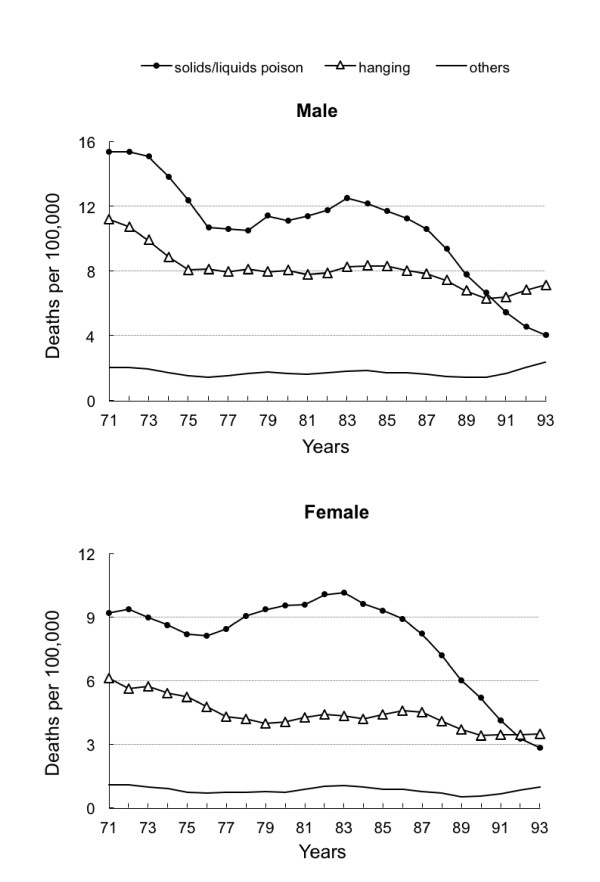
**Three-year moving average of age-adjusted suicide rates by methods in Taiwan, 1971-1993**.

**Figure 2:**
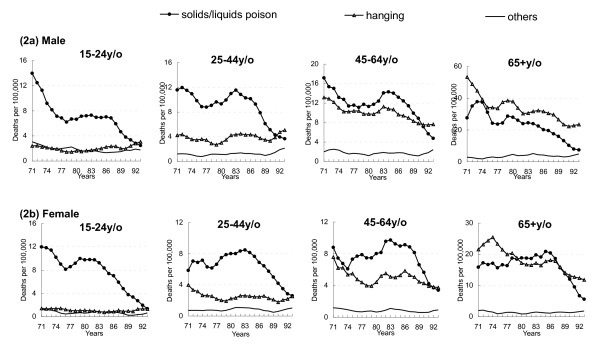
**Three-year moving average of age-adjusted suicide death rates by method and age in Taiwan, 1971-1993**.

Figure 1 represents the age-adjusted method-specific suicide trends with 3-year moving average for both sexes, from 1971 through 1993 in Taiwan. During the study periods, solids/liquids poisoning and hanging were the two major suicide methods. The solids/liquids suicide rates markedly and steadily decreased from 12.6 per 100,000 to 3.7 in males and from 10.0 to 2.6 in females between 1983 and 1993. Around the same time, there was no obvious rise in the suicide rates for hanging or other methods in both sexes. Further analysis was done by examining method-specific suicide trend by age and sex from 1971-1993 and found that limited substitution seemed to takes place among males aged 25-44 years (Figure 2).

Table 2 clearly illustrates that there were significant downward slopes in the sex-specific suicide rates by solids/liquids during the period of 1983-1993 in Taiwan. The slope for solids/liquids suicide rates was -0.98 (i.e., an annual decrease in the solids/liquids suicide rates of 0.98 per 100,000) (95% CI = -1.12, -0.80) for males and -0.82 (95% CI = -0.96, -0.67) for females. The slope for hanging suicide rates was also downward, i.e., -0.20 (95% CI = -0.36, -0.04) for males and -0.13 (95% CI = -0.21, -0.04) for females. Although the slope for suicide rates by other methods was upward, the value was very small and with no statistical significance, i.e., 0.07 (95% CI = -0.01, 0.15) for males and 0.004 (95% CI = -0.04, 0.05) for females. Significant downward slopes for the sex-specific solids/liquids suicide rates during the period 1983-1993 in Taiwan were also found among the four age groups (see Table 2).

**Table 2:** Linear regression slopes of age-adjusted method-specific suicide rates by sex and age in Taiwan, 1983-1993

	Male				Female			
	
	**Coeffi**.	SE	t	p-value	**Coeffi**.	SE	t	p-value
15-24 y/o								
Poisoning^a^	-0.60	0.07	-8.17	0.000***	-0.74	0.05	-14.69	0.000***
Hanging^b^	0.09	0.05	1.75	0.114	0.04	0.03	1.29	0.228
Others^c^	0.06	0.03	2.19	0.056	0.00	0.02	-0.12	0.907
25-44 y/o								
Poisoning^a^	-0.86	0.10	-8.94	0.000***	-0.65	0.05	-14.35	0.000***
Hanging^b^	0.07	0.07	0.96	0.360	-0.01	0.04	-0.27	0.795
Others^c^	0.09	0.04	2.39	0.041*	0.01	0.03	0.31	0.765
45-64 y/o								
Poisoning^a^	-1.16	0.08	-13.87	0.000***	-0.73	0.12	-6.07	0.000***
Hanging^b^	-0.42	0.10	-4.12	0.003**	-0.19	0.06	-3.02	0.014*
Others^c^	0.07	0.05	1.32	0.218	-0.01	0.02	-0.35	0.734
65 above								
Poisoning^a^	-1.86	0.22	-8.43	0.000***	-1.86	0.19	-9.88	0.000***
Hanging^b^	-1.30	0.22	-5.81	0.000***	-0.77	0.15	-5.17	0.001***
Others^c^	0.05	0.12	0.38	0.713	0.05	0.03	1.82	0.102
Total								
Poisoning^a^	-0.98	0.08	-12.45	0.000***	-0.82	0.06	-13.09	0.000***
Hanging^b^	-0.20	0.07	-2.84	0.019*	-0.13	0.04	-3.29	0.009**
Others^c^	0.07	0.04	2.05	0.071	0.00	0.02	0.18	0.858

^a ^Poisoning by solid or liquid substances

^b ^Hanging, strangulation and suffocation

^c ^Others including poisoning by gases, drowning, firearms, cutting and piercing, jumping/falling from high places, other and unspecified means and late effects of injury

p-value: * < 0.05; ** < 0.01; *** < 0.001

Table 3 shows the changes in method-specific suicide rates by sex and age between 1983 and 1993. Among the four age groups, a marked decrease (a 69% to 86% decrease) in the solids/liquids suicide rates contributed to the reduction of overall suicide rates (about a 39% to 67% decline). In addition, no obvious compensatory rise in suicide rates by hanging or other methods accompanied the great decrease in the solids/liquids suicide rates found between 1983 and 1993.

**Table 3:** Changes in method-specific suicide death rates by sex and age between 1983 and 1993 in Taiwan

	Male					Female				
	
	1983		1993		Rate Difference	1983		1993		Rate Difference
	Rate	N	Rate	N		Rate	N	Rate	N	
15-24 y/o										
Poison^a^	7.18	(147)	1.9	(36)	-5.32	7.57	(149)	1.03	(19)	-6.55
Hanging^b^	2.03	(41)	2.6	(51)	0.61	0.73	(15)	1.03	(19)	0.3
Others^c^	1.52	(32)	1.8	(35)	0.27	0.47	(9)	0.8	(15)	0.33
Overall	10.7	(220)	6.3	(122)	-4.44	8.77	(173)	2.86	(53)	-5.92
25-44 y/o										
Poison^a^	10.5	(268)	3.1	(111)	-7.39	9.05	(235)	2.61	(88)	-6.44
Hanging^b^	3.91	(102)	4.5	(163)	0.597	2.3	(57)	2.58	(91)	0.28
Others^c^	1.39	(38)	2	(74)	0.625	1.04	(27)	1.18	(40)	0.14
Overall	15.8	(408)	9.7	(348)	-6.17	12.4	(319)	6.36	(219)	-6.03
45-64 y/o										
Poison^a^	15.8	(255)	5	(84)	-10.9	9.43	(117)	2.98	(49)	-6.45
Hanging^b^	13.1	(214)	7.3	(125)	-5.8	4.09	(52)	3.7	(60)	-0.39
Others^c^	1.67	(28)	3	(52)	1.33	1	(12)	0.81	(13)	-0.19
Overall	30.6	(497)	15	(261)	-15.4	14.5	(181)	7.5	(122)	-7.02
65 above										
Poison^a^	24.5	(109)	7.3	(60)	-17.2	20.8	(89)	5.26	(37)	-15.56
Hanging^b^	33.9	(148)	21	(174)	-12.4	17.1	(75)	10.4	(73)	-6.69
Others^c^	4	(19)	5.1	(43)	1.084	1.52	(6)	1.57	(10)	0.05
Overall	62.4	(276)	34	(277)	-28.6	39.5	(170)	17.3	(120)	-22.2
Total										
Poison^a^	12.6	(779)	3.7	(291)	-8.84	9.99	(590)	2.59	(193)	-7.4
Hanging^b^	9.01	(505)	6.5	(513)	-2.48	3.91	(199)	3.3	(243)	-0.61
Others^c^	1.77	(117)	2.5	(204)	0.778	0.94	(54)	1.02	(78)	0.09
Overall	23.4	(1401)	13	(1008)	-10.5	14.8	(843)	6.92	(514)	-7.91

^a ^Poisoning by solid or liquid substances

^b ^Hanging, strangulation and suffocation

^c ^Others including poisoning by gases, drowning, firearms, cutting and piercing, jumping/falling from high places, other and unspecified means and late effects of injury

In further analysis of the age-adjusted poisoning suicide rates by pesticides, medications, and other substances for the years 1987 and 1992, we found that as the male pesticide suicide rates decreased from 5.6 to 2.1 per 100,000 from 1987 to 1992, the medication suicide rates and other substance suicide rates also decreased, from 1.4 to 1 per 100,000 and from 1 to 0.4 per 100,000, respectively. For females, the age-adjusted suicide rates by pesticides, medications, and other substances also decreased from 1987 to 1992: pesticide suicide rates decreased from 3.6 to 1.7 per 100,000, and medication suicide rates decreased 1.3 to 0.6 per 100,000; other substance suicide rates decreased from 1 to 0.5 per 100,000.

## Discussion

Our findings indicated no compensatory rise in suicide rates by hanging or other methods from 1983 to 1993 in Taiwan, accompanying the marked decrease in solids/liquids poisoning suicide rates. The same pattern of change occurred in different sex and age groups.

Several previous studies illustrated that restricting access to lethal methods can reduce suicide rates [1-11]. The most often cited example was Kreitman's study [1], which demonstrated a reduction not only in gas-specific suicide rates, but also in the overall suicide rates because of the lack of a compensatory rise in the use of other methods. However, Gunnell et al., who offered the criticism that those analyses have either failed to examine trends in method-specific suicide rates or have not assessed age- and gender-specific effects, reanalyzed the suicide trends in England and Wales in 1950-75, and found that the effects of these reductions on overall suicide rates due to coal gas detoxification were partially offset by rises in drug overdose deaths in women and younger men [13].

In fact, some other studies showed that method substitution did occur in some specific subgroups and with certain methods, as summarized in Table 1[3,8,12-15]. Among those studies, method substitution seemed to be most likely found in young people [14,15] or in males [3,8,12]. In addition, as seen in Table 1, we also found that non-violent methods (e.g., domestic gas) were often replaced by non-violent methods (e.g., car exhaust or drug overdose) [3,12,13] and that violent methods (e.g., firearms) were often replaced by violent methods (e.g., jumping or hanging) [8,14].

If suicide rates in Taiwan followed the substitution hypothesis, we would expect a compensatory increase in suicide rates by overdose, domestic gas and other gas in specific demographic subgroups. Our analyses, however, did not find such results. Why was there no method substitution in Taiwan? And what are the factors affecting the occurrence of substitution?

Three possible factors proposed by Gunnell et al. [13], i.e., the accessibility to, and the acceptability and popularity of the lethal methods were found to have profound effects on method substitution.

As to accessibility, there were nearly no limitations on the availability of medications, since people in Taiwan were free to visit physicians and to buy medications in the drugstore. Similarly, domestic gas was also available to nearly all people, because it was the main heating source for cooking and bathing in every household. Also, car ownership had increased annually due to the 1983-1993 economic development in Taiwan.

In terms of acceptability, there was no stigma to using poisoning with medications, poisons and gas as suicide methods in Taiwan society. However, there was still no method substitution in Taiwan. This may be due to the lack of popularity of other suicide methods during the period 1983-1993 in Taiwan.

The most popular suicide methods in Taiwan were poisoning with solids/liquids (mainly pesticides) and hanging, which constituted 90% of all suicide deaths in the period 1983-1993 [18]. The other suicide methods popular in Western countries, such as car exhaust, medicine overdose, and gassing, contributed little to suicide deaths in Taiwan. Therefore, the lack of popularity of other violent suicide methods similar to pesticide overdosing before 1993 may be one of the possible explanations for the phenomenon of no method substitution in Taiwan.

There were some limitations to our study. First, the E-codes of the ICD-8 and ICD-9, used in Taiwan during the study periods, only included three-digits. The code E950 (and its analogous counterpart E980) is a broad category that includes suicide from all types of solid and liquid substances, such as pesticides, prescriptions and other poisons. However, the data from the years 1987 and 1992 show that pesticides were the major ingested solid/liquid substances causing suicide death in Taiwan. In addition, no compensatory rise in suicide rates by medications and other solid/liquid substances was found, as the pesticide suicide rates decreased markedly from 1987 to 1992. In fact, the sex-specific suicide rates by poisoning with pesticides, medications and other substances all decreased from 1987 to 1992.

Second, suicide death is thought to be a complex phenomenon involving multiple factors. The influence of many other factors, such as mental health and the socio-economic environment on sex-, age- and method-specific suicide rates was not considered in this study. Further study is needed to test whether changes in psycho-socio-economic conditions would have differential effects on the method-specific suicide rates among particular subpopulations.

Thirdly, one may wonder why did authors not include more recent years for analysis? In fact, the suicide methods changed much in the recent decade in Taiwan. In a previous study, Lin and Lu reported [21], during the 1970's and 1990's, the two leading suicide methods in Taiwan were poisoning by solids/liquids and hanging. These two methods were responsible for about 90% of all suicide deaths before 1990. In the contemporary years of 1991-2005, nearly all method-specific suicide rates seemed to increase in both sexes. However, the distribution of suicide methods changed much. The contribution of the traditional suicide methods in Taiwan (i.e., hanging and poisoning by solids/liquids) decreased, while that of the new methods (i.e., jumping from heights and poisoning with other gases) markedly increased. In this study, we focused on the testing of substitution hypothesis. So, we chose the period 1983-1993, when the suicide methods were relatively simple, to test the substitution hypothesis.

### Implications for suicide prevention

As Gunnell et al estimated that pesticide self-poisoning accounted for about one-third of the world's suicides and suggested that we might prevent many of these deaths by restricting the access to pesticides [22]. Our results add to the evidence that restricting access to lethal methods is a promising strategy in suicide prevention, particularly in those countries where the "target method" contributed greatly to the suicide rate. Moreover, when we plan to restrict one lethal suicide method, we should also be aware of the popularity of other possible substitute methods with equal access and similar acceptability.

## Conclusion

In general, no method substitution was found along with the reduction in solids/liquids suicide rates in Taiwan. Our study results have also added the evidence that restricting access to methods maybe a promising strategy in preventing suicide, particularly in those countries where the "target method" has been found to contribute greatly to the suicide rates.

## Competing interests

The authors declare that they have no competing interests.

## Authors' contributions

JJL contributed to the study design, analysis and interpretation of the data and drafted the paper. THL contributed to the study design, obtained the data and commented on the interpretation. All authors have read and approved the final manuscript.

## Pre-publication history

The pre-publication history for this paper can be accessed here:

http://www.biomedcentral.com/1471-2458/11/712/prepub
